# Open Source Automated Flow Analysis Instrument for Detecting Arsenic in Water

**DOI:** 10.1016/j.ohx.2022.e00284

**Published:** 2022-03-09

**Authors:** Julián Gutiérrez, Juan Pablo Mochen, Gabriel Eggly, Marcelo Pistonesi, Rodrigo Santos

**Affiliations:** a Departamento de Ingeniería Eléctrica y de Computadoras - ICIC, Universidad Nacional del Sur-CONICET; b Departamento de Química-INQUISUR, Universidad Nacional del Sur - CONICET, CIC PBA; c Departamento de Ingeniería Eléctrica y de Computadoras - ICIC, Universidad Nacional del Sur - CIC PBA

**Keywords:** Piezoelectric, QCM, Arsenic, Flow-Batch, analyser

## Abstract

In this paper the design and implementation of an embedded system based on Flow-Batch methodology with a Quartz Crystal Microbalance (QCM) sensor technology and a commercial FPGA admittance meter is presented to detect the presence of arsenic in water. The system’s performance was evaluated with lab made samples and it is foresee that this open source automated flow instrument could help develop analytical methodologies for the future quantification of this analyte. A description of the components is presented and assembling and operation instructions are provided together with the dynamic range and linear regression coefficients for the line and R.

## Graphical abstract

**Figure:**
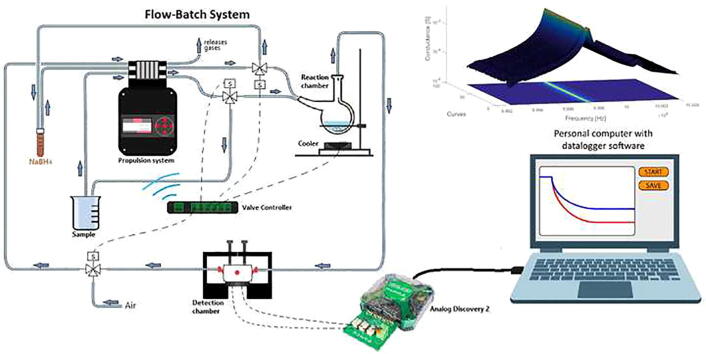

## 1 Hardware in context

Most of the analytical methods for arsenic, including field methods, take advantage of the formation of volatile arsine (${\mathrm{AsH}}_{3}$) gas to separate the arsenic from other possible interferences in the sample matrix. For over a half century, the Gutzeit generator was the only well recognized pretreatment method for the determination of trace quantities of arsenic. Sodium borohydride selectively reduces arsenic to arsine from the sample buffered at pH 5-6. The generation of the arsenic hydride and the formation of excess hydrogen gas as a carrier gas have to continue for long enough to expel all of the arsenic from the sample, yet be sufficiently rapid to enable a reasonable sample throughput. QCM is a special quartz crystal arranged such that its resonant frequency (i.e., its electrical admittance) changes as a function of chemicals adsorbed or doped on its surface. As gaseous arsine flows into the chamber, the density of the gas over the QCM changes and it is reflected into variations in the resonating frequency. There are several works in the literature that described the use of QCM sensors like [1], [2].

**Table:** 

**Specifications table**		

**Hardware name**	Analyser to determine arsenic in water	

**Subject area**		
	•*Chemistry and biochemistry*	
	•*Environmental, planetary and agricultural sciences*	
	•*General*	

**Hardware type**	•*Measuring physical properties and in-lab sensors*	
	•*Field measurements and sensors*	
	•*Electronic engineering and computer science*	

**Closest commercial analog**	No commercial analog is available.	

**Open source license**	CERN OHL v1.2	

**Cost of hardware**	Two thousand and one hundred dollars	

**Source file repository**	doi:10.17632/499gx3pfxk.1	

The whole control of the Flow-Batch system and the gaseous flow is handled with a microcontroller platform based on the ESP-32 device while the admittance measurement is done with a commercial FPGA instrument. The system was built in the lab using several commercial elements like the solenoid valves or stepper motors. Beaker, reaction and measurement chambers were built in the lab, like the PCB and the assembly of the electronics together with different pieces specially design and built with a 3D printer for the peristaltic pump and several other elements. Other methods proposed in the literature to determine arsenic can be found in [3], [4], [5].

## 2 Hardware description

**Fig. 1:**
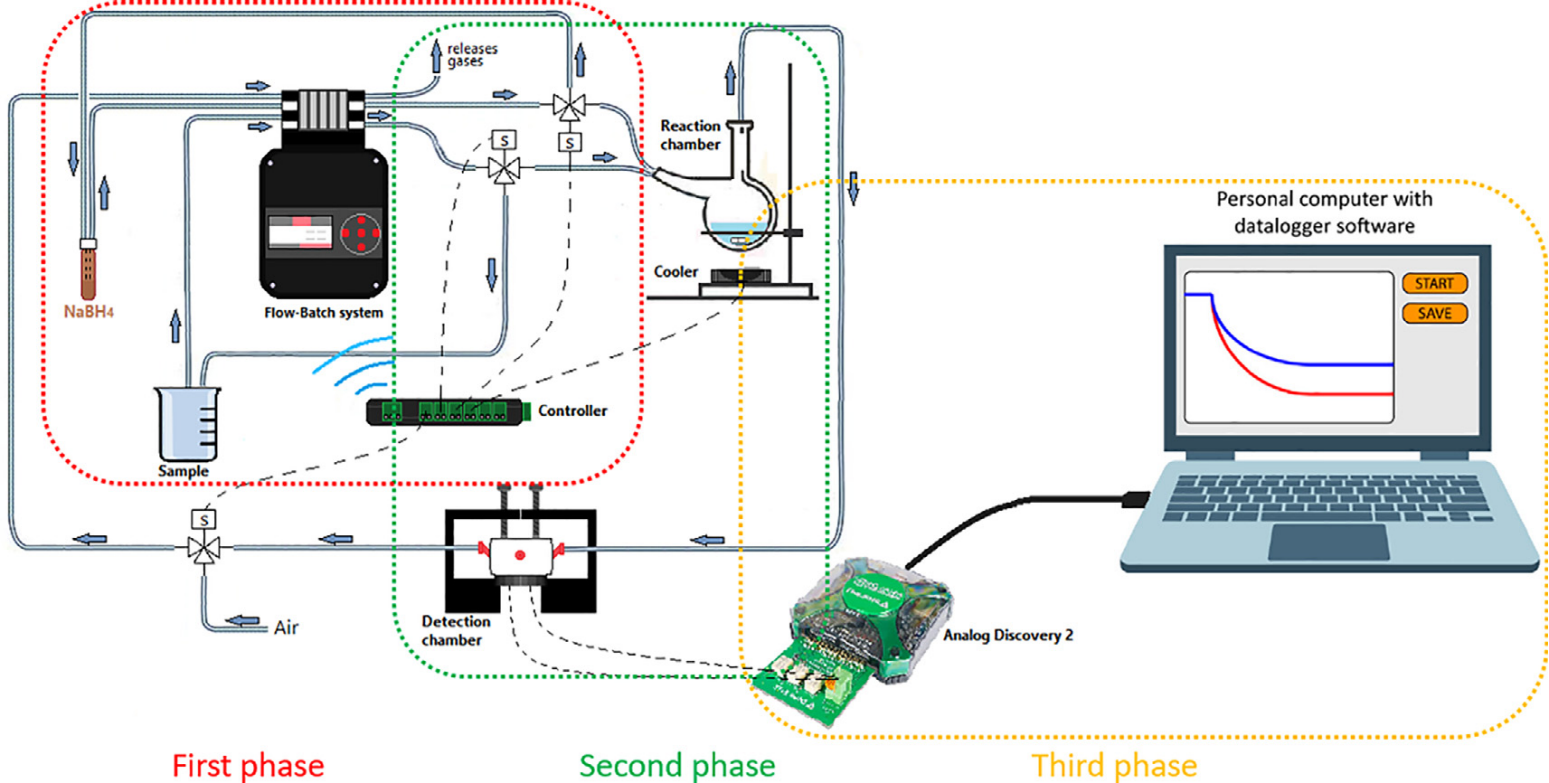
Flow-Batch system.

The proposed system, which can be seen in Fig. 1 as a block diagram, can be described in its component parts to carry out the process of taking and preparing the sample, and measuring the analyte.

The Flow-Batch system consist of two subsystems: one of these is in charge of the propulsion and control of fluids, and the other is in charge of the sample reaction and analyte detection. The first is the Flow-Batch controller. This sub-system consists of the pumps and valve actuator. Together they propel and direct fluid to different parts of the instrument.The second part is the detection sub-system. This consists of two chambers: a reaction chamber for producing the arsine gas from the sample and a detection chamber that holds the QCM in which the measurement is performed. The response of the QCM sensor admittance variation is performed with a special FPGA circuit that contains an Impedance Analyzer Module connected directly to the sensor. This analyzer registers the variations in the admittance and provides an output to be processed later with Octave software.

A complete description of the operation of the flow-batch system is provided in 6, 7. As can be seen in Fig. 1, there are three phases in the measurement process. The first one prepares the sample to be analysed. In this phase, the components are circulating with the peristaltic pump while the solenoid valves are closed. Once the system is ready, the solenoid valves are activated and the components are mixed in the reaction chamber. This second phase produces the chemical reaction where the analyte is processed for its separation and detection through (in this case) the QCM sensor that registers the presence of the arsenic. In the third and last phase, the data register through the QCM sensor and FPGA Impedance Analyzer Module is processed using Octave software and the result of the measurement is obtained. The three phases are necessary for the Flow-Batch system to operate.

In what follows a more detail description of the different parts is provided with the component selection and configuration.

### 2.1 Flow-Batch Controller

This section details the development and construction of a Flow-Batch controller prototype for various applications in analytical chemistry where this methodology can be applied.

The Flow-Batch controller is composed by two separate units, the propulsion system and the valve actuator. The propulsion system is assembled with a custom drive unit and a commercial pump head with four channels (Pump Head R4 for Gilson minipulse).

The drive unit acts as the master controller of the Flow-Batch controller. It sends commands through a WiFi protocol (ESP-NOW) to the valve actuator for the activation of the outputs, and thus, activating the corresponding valve for the time configured.

The main characteristics of this system are:

•Propulsion system capable of varying from 0 to 50 rpm with user-friendly interface to configure all the application parameters of a Flow-Batch system.
•Wireless Valve activator with 12V digital outputs for the multi-commutation system.

#### 2.1.1 Propulsion system

A fundamental characteristic in peristaltic pumps is that the speed of the motor can be controlled so that, in this way, the resulting flow is proportional to this speed. One point to keep in mind is that the torque can vary depending on the speed. Stepper motors (PaP) are electromechanical devices that convert a sequence of electrical impulses into discrete angular displacements. This allows very precise control of rotation without the need for feedback. They also maintain their maximum torque throughout most of their speed range.

To move the head of the peristaltic pump, a high torque Nema 17 bipolar PaP motor with a 5:1 reducer coupled to the shaft was chosen. This makes it possible to further increase the torque, and in turn, smooth the discreet movement of this type of motor.

In the selection of the hardware platform, some aspects were taken into account such as: availability in the local market, good cost-performance ratio, robustness, wireless communication capacity, and finally, that the number of available I/O ports were sufficient to interact with all the devices in the system. Under these restrictions we have chosen a nodeMCU platform, based on the ESP-32s microcontroller, which satisfies all the mentioned requirements.

In order to efficiently govern the motor from the control unit, a Pololu DRV8825 driver was used. This board features a TI-developed DRV8825 microstepping bipolar stepper motor driver. It features adjustable current limiting, overcurrent and overtemperature protection, and six microstep resolutions (up to 1/32-step).

The system can be powered in the range of 12 to 40VDC. It has a buck converter LM2596 to regulate the output voltage at 5V for the microcontroller. The use of a peristaltic pump and the associate solenoid valves in this system is necessary to keep a constant flow of the analyte over the QCM detection sensor. In some other systems the used of metering pumps may be an option if the perturbations coming from the pulses introduced by this kind of pumps do not interfere with the detection or determination process.

**Fig. 2:**
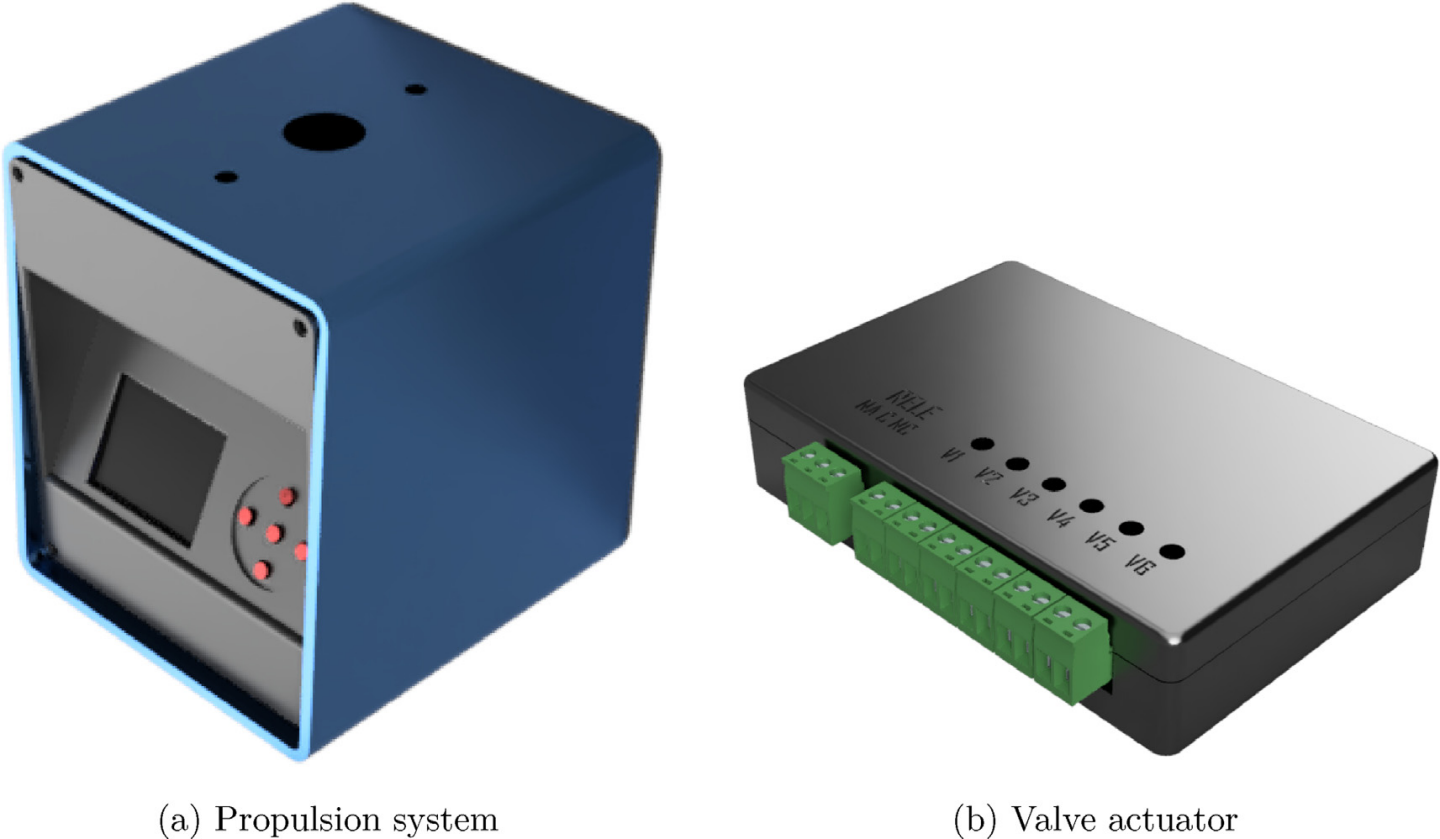
Flow-Batch controller.

For the interaction with the system, it has an HMI interface composed of a 2.4 inches ILI9341 TFT display and 5 buttons for navigation between menus and configuration of system parameters. Fig. 2 shows the prototype developed.

#### 2.1.2 Valve actuator

The most commonly found actuators in FB assemblies are solenoid valves and stirrers made with direct current (DC) motors. To be able to command this type of actuator from the control unit, a circuit board was built consisting of an ESP8266 microcontroller and an arrangement of power transistors that condition the low intensity digital signals (from the CMOS, TTL logic gates, etc.).

To simplify the design, the ULN2803A integrated circuit was used, which has an array of 8 Darlington transistors with common emitter and damper diodes for inductive loads. Each Darlington can deliver a continuous current of 500 mA (600 mA peak) and supports a voltage of up to 50V.

The controller is powered by a 3.7V lithium battery which can be recharged with a phone charger through the TP4056 module included on the PCB. The battery voltage is raised from 3.7V to 12V (required for actuators) through the MT3608 boost converter. To power the microcontroller, an LM1117-5 regulator lowers the voltage from 12V to 5V.

### 2.2 Reaction and detection chamber

The process by which a sample of water is transformed into a gaseous flow is controlled by the Flow-Batch system that uses the controller (described in previous section), a reaction chamber to contain a chemical reaction and finally, a detection chamber containing the QCM sensor.

#### 2.2.1 Reaction chamber

**Fig. 3:**
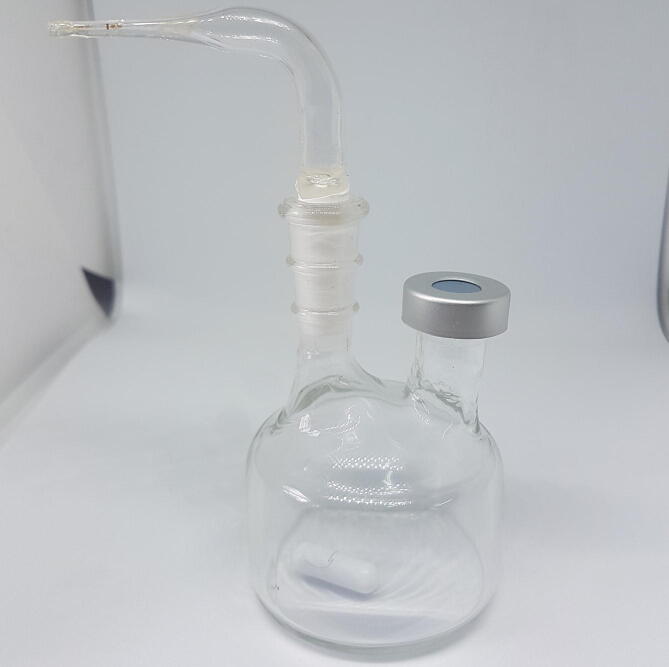
Flask used as reaction chamber.

In this method, arsine is generated by the reduction of inorganic arsenic in the water sample, using a sodium borohydride solution. The reaction is carried out in a glass flask designed in a glassblowing workshop. This custom glass vessel consists of a 20 mL custom two-neck round bottom flask with an inlet for liquid solution (through rubber septa) and an outlet for gases. The solutions inside the reaction chamber are homogenized with a magnetic stirrer (developed with an electronic cooler with a magnet) and a stir bar inside the chamber. The reaction chamber can be seen in Fig. 3. The stirrer can be seen in the bottom of the flask.

To construct the system without a glassblower workshop, an equivalent chamber could be built in Teflon using a lathe.

#### 2.2.2 Detection chamber

The gas produced comes out of the reaction chamber towards the detection chamber. The detection chamber consists of two parts: the top and the bottom part.

The top part of the chamber is built of a solid cylindrical piece of Teflon of 25 mm height with 40 mm in the external diameter and 12 mm in the internal diameter. The depth inside is 14 mm and has an internal volume of 1.5 mL. It has four channels to move the fluids through it. A Teflon washer was incorporated to reduce the internal diameter from 12 to 6 mm to adjust it with an o-ring. Teflon is a hydrophobic material which is important as the chamber can be clean with water without leaving residues.

The bottom piece was designed and built using a 3D printer to hold all the parts necessary for the analyser device. The piece is designed to couple with the o-ring of the Teflon chamber. To hold the QCM, a mould with its dimensions was built and two electrical contacts included in the electrodes positions. These contacts connect the QCM with the driving circuit.

**Fig. 4:**
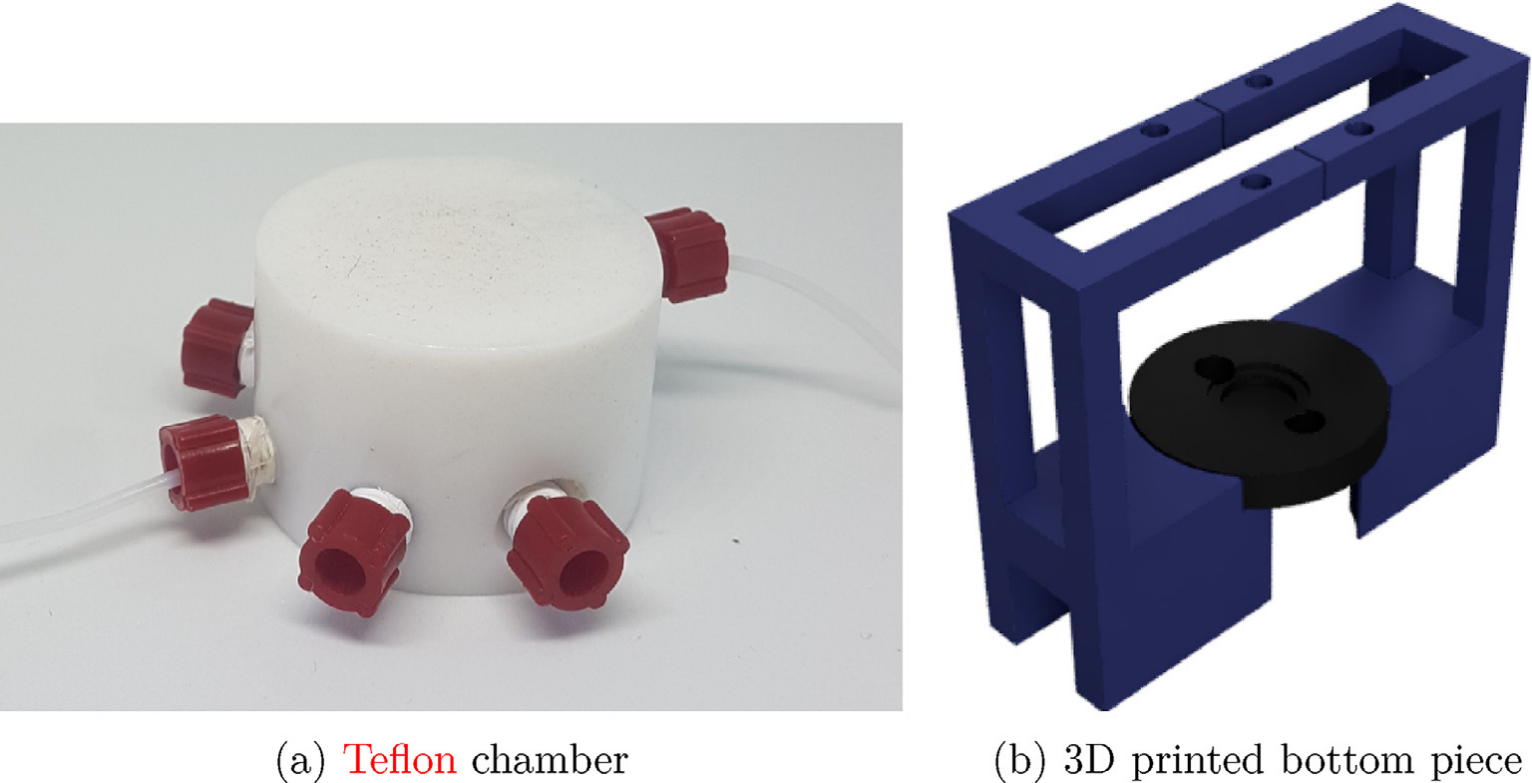
Detection chamber.

All the parts are held together with appropriate screws and nuts. Fig. 4, Fig. 4b show the chamber and how it is assembled together with the 3D piece, respectively.

### 2.3 Admittance measurement

**Fig. 5:**
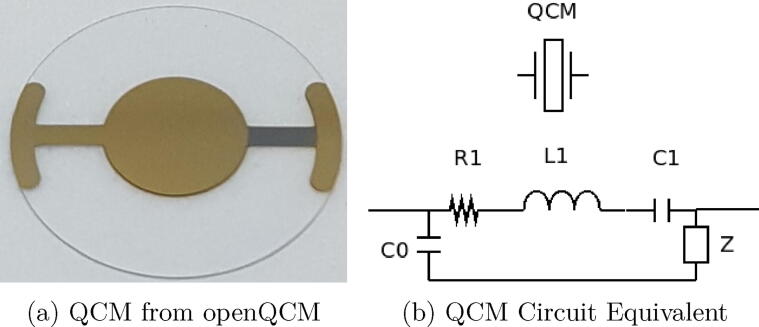
QCM.

QCM is a special kind of piezoelectric that changes its resonating frequency according to the presence of different elements on its surface. Fig. 5 shows the QCM used in the system. The doping process is controlled with the Flow-Batch controller. The matrix to be analysed is pre-treated in such a way that the desired element to measure its concentration is isolated. In this case, the analyser is designed to determine the concentration of arsenic in water. The arsenic is determined through the presence of a gaseous arsine flow. In the reaction chamber, arsine will be generated which will then pass to the measurement chamber through a continuous flow. From the changes in densities, variations in the QCM parameters can be obtained without the need for the use of a reagent. We selected the QCM sensor from OpenQCM company for our instrument. It is a quartz crystal with 13.9 mm of diameter and 160 *u*m thickness with a natural oscillating frequency in 10 MHz. The effective area is composed by two gold electrodes (one on each side) with each one having a 6 mm diameter. These are the contacts through which the electrical connection is made to the electronic circuit. It has a sensitivity of 4.42 $\frac{\mathrm{ng}}{\mathrm{Hz}.{\mathrm{cm}}^{2}}$ with a stability of ± 20 KHz at $23^{o}C$.

The electronic equivalent circuit of the QCM is shown in Fig. 5b and is based on the model proposed by Butterworth-Van Dyke in which Z changes according to the variations in the doped surface of the QCM [6]. The admittance of an element indicates how much current is allow to flow through the device or what is the same the inverse of the impedance.

The admittance can be computed as

$$Y=\frac{1}{R_{1}+j\omega L_{1}-\frac{1}{j\omega C_{1}}+Z}+j\omega C_{0}$$

where R,L,C are the values of resistance, inductance and capacitance respectively and jω indicates the complex term associated with the electrical expression.

**Fig. 6:**
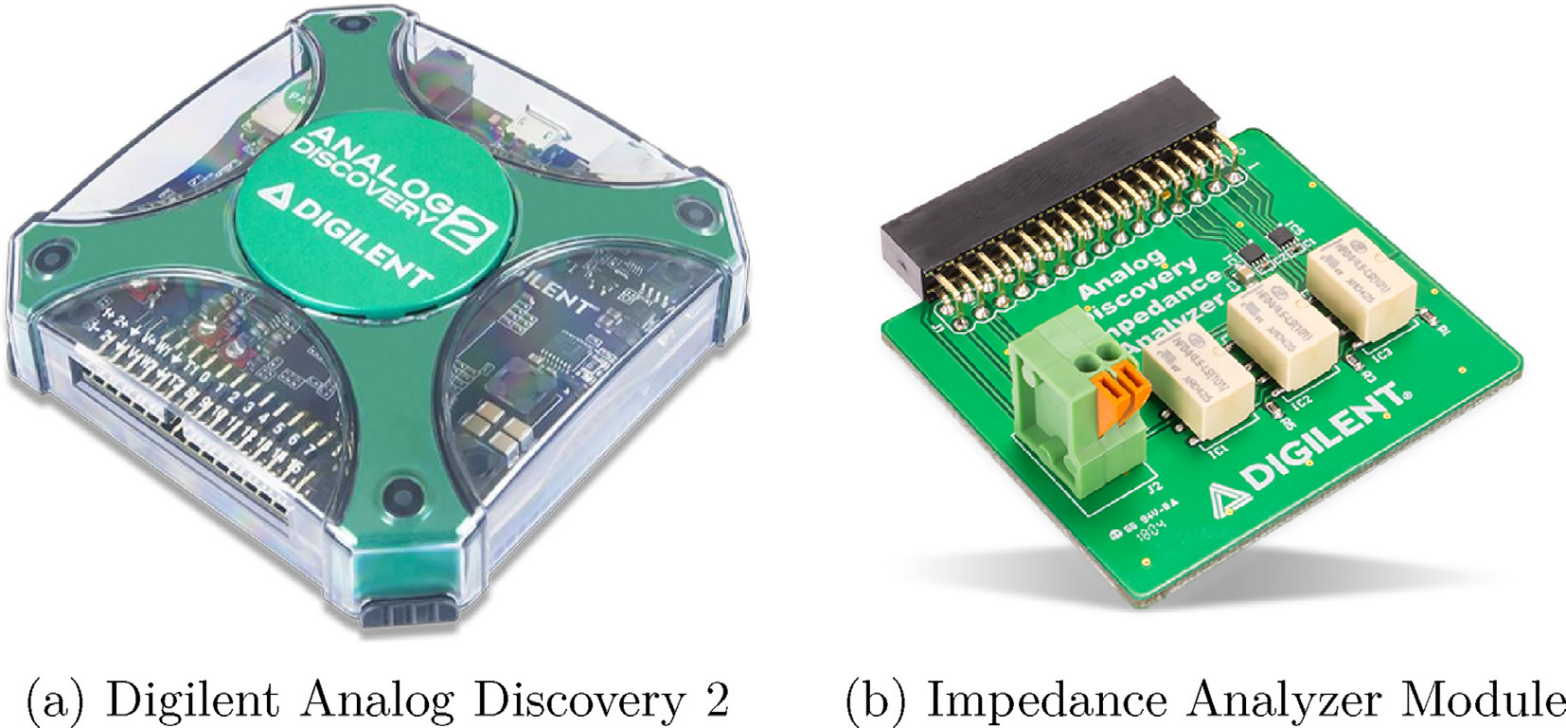
Impedance Analyzer.

The admittance variations modified the resonating frequency of the crystal but these variations should be measured with special devices. In this system, a commercial FPGA circuit was selected to do it. The Digilent Analog Discovery 2, Fig. 6a, is a multifunction instrument that can visualize, generate, register and control mixed signal circuits of different kind. It works as logic analyzer and oscilloscope. It has enough processing power to replace several instruments when the professional has to work out of the lab with sufficient precision. The FPGA has the possibility to incorporate an external accessory, like an impedance analyzer to perform the admittance analysis variation method, Fig. 6b. With this instrument a variation in the admittance of the QCM can be measured quite simply. The electrodes should be properly connected to the FPGA device.

## 3 Design files summary

### 3.1 Electronics

The schematics and PCB designs were created in EasyEDA CAD software. From the same software environment, you can place the order for the manufacturer of the PCB.

### 3.2 3D Parts

All 3D-printed parts were created using fusion360 CAD software in order to leverage the parametric nature of the platform and enhance flexibility of the design. All individual parts were exported as STL files for further manufacturing. Parts were fabricated using a 3D printer and PLA filament configured with 0.2 mm layer height.

### 3.3 Software

The Flow-Batch controller software was developed using the Arduino IDE. It consist of two folders for Flow-Batch controller and valve actuator. Arduino IDE has to be configured to program the ESP-32 and ESP8266 microcontrollers.

WaveForms is the free software application for the Analog Discovery 2. The FPGA communicates with WaveForms via USB connection to the computer, allowing users to capture, record, analyze, and generate mixed signal and mixed domain waveforms. In addition to the use of instruments in the application, the software has a script editor tool, which allows custom scripting of the instrument in JavaScript.

**Table:** 

			

**Design filename**	**File type**	**Open source license**	**Location of the file**

Part #1 - PCB_FlowBatch_Controller.json	PCB design file	CERN OHL v1.2	doi:10.17632/499gx3pfxk.1

Part #2 - PCB_HMI-Panel.json	PCB design file	CERN OHL v1.2	doi:10.17632/499gx3pfxk.1

Part #3 - PCB_Valve_Controller.json	PCB design file	CERN OHL v1.2	doi:10.17632/499gx3pfxk.1

Part #4 - FBS_NemaHolder.stl	3D Printing file	CERN OHL v1.2	doi:10.17632/499gx3pfxk.1

Part #5 - FBS_PumpHeadSupplement.stl	3D Printing file	CERN OHL v1.2	doi:10.17632/499gx3pfxk.1

Part #6 - FBS_Case.stl	3D Printing file	CERN OHL v1.2	doi:10.17632/499gx3pfxk.1

Part #7 - FBS_HMI-Panel.stl	3D Printing file	CERN OHL v1.2	doi:10.17632/499gx3pfxk.1

Part #8 - FBS_Buttons.stl	3D Printing file	CERN OHL v1.2	doi:10.17632/499gx3pfxk.1

Part #9 - FBS_BackPanel.stl	3D Printing file	CERN OHL v1.2	doi:10.17632/499gx3pfxk.1

Part #10 - VC_Case_Bottom.stl	3D Printing file	CERN OHL v1.2	doi:10.17632/499gx3pfxk.1

Part #11 - VC_Case_Top.stl	3D Printing file	CERN OHL v1.2	doi:10.17632/499gx3pfxk.1

Part #12 - MS_Case_Bottom.stl	3D Printing file	CERN OHL v1.2	doi:10.17632/499gx3pfxk.1

Part #13 - MS_Case_Top.stl	3D Printing file	CERN OHL v1.2	doi:10.17632/499gx3pfxk.1

Part #14 - GC_Low_Side_Chamber.stl	3D Printing file	CERN OHL v1.2	doi:10.17632/499gx3pfxk.1

Part #15 - GC_Press_A.stl	3D Printing file	CERN OHL v1.2	doi:10.17632/499gx3pfxk.1

Part #16 - GC_Press_B.stl	3D Printing file	CERN OHL v1.2	doi:10.17632/499gx3pfxk.1

Part #17 - flowbatch_controller.ino	Software	GPL	doi:10.17632/499gx3pfxk.1

Part #18 - valve_controller.ino	Software	GPL	doi:10.17632/499gx3pfxk.1

Part #19 - admittance_meas.js	Software	GPL	doi:10.17632/499gx3pfxk.1

FlowBatch_Controller.f3d	CAD file	CERN OHL v1.2	doi:10.17632/499gx3pfxk.1

FlowBatch_Chambers.f3d	CAD file	CERN OHL v1.2	doi:10.17632/499gx3pfxk.1

Stirrer_Case.f3d	CAD file	CERN OHL v1.2	doi:10.17632/499gx3pfxk.1.

FlowBatch_Controller.json	Schematic file	CERN OHL v1.2	doi:10.17632/499gx3pfxk.1

The listed design files contain all the necessary information to reproduce the hardware. All the 3D printing files contained the models for the 3D printer to produce the pieces of the Flow-Batch system. In particular the chambers, panels, cases, and buttons. The CAD files complement the 3D printing files. The json files contained the PCB description and schematics as generated with the EasyEDA design software. Finally, the ino files correspond to the firmware generated for the microcontrollers to handle the whole Flow-Batch and valve control processes.

## 4 Bill of materials Summary

**Table:** 

							

**Designator**	**Component**	**Number**	**Cost per unit- USD**	**Total cost- USD**	**Source of materials**	**Material type**	

PCB Parts	Electronic components for soldering on PCBs	1		$85.29	DIGIKEY	Semiconductor	

Part #20	IDC Connector 16 Position Rectangular Header Gold 28 AWG	2	$0.79	$1.58	DIGIKEY	Other	

Part #21	Flat Ribbon Cable Gray 16 Conductors 0.050”	1	$0.84	$0.84	DIGIKEY	Other	

Part #22	Battery Holder 18650 1 Cell	1	$3.11	$3.11	DIGIKEY	Other	

Part #23	13.7 V Lithium-Ion Battery Rechargeable 2.6Ah	1	$4.99	$4.99	DIGIKEY	Other	

Part #24	High Torque Planetary Gear 5:1 Motor Nema17	1	$40.05	$40.05	AMAZON	Metal	

Part #25	Pump Head R4 for Minipulse	1	$1,108.00	$1,108.00	GILSON	Metal and Polymer	

Part #26	Flexible shaft coupler	1	$6.49	$6.49	AMAZON	Metal	

Part #27	PVC Pump Tubing, id 0.76mm (black-black)	1	$48.46	$48.46	GILSON	Polymer	

Part #28	PVC Pump Tubing, id 1.70mm (gray-gray)	1	$48.46	$48.46	GILSON	Polymer	

Part #29	PVC Pump Tubing, id 2.06mm (purple-purple)	1	$48.50	$48.50	GILSON	Polymer	

Part #30	Tygon tubes	1	$14.42	$14.42	GILSON	Polymer	

Part #31	PLA filament 1.75mm, 1Kg	1	$19.59	$19.59	AMAZON	Polimer	

Part #32	QCM 10MHz	1	$15.90	$15.90	openQCM	Other	

Part #33	Digilent Analog Discovery 2	1	$399.00	$399.00	DIGILENT	Semiconductor	

Part #34	Impedance Analiser for Analog Discovery 2	1	$21.99	$21.99	DIGILENT	Semiconductor	

Part #35	3-Ways Solenoid Valve, 12VDC	3	$70.00	$210.00	COLE PARMER	Metal and Polimer	

The full version of the Bill of Materials that contains electronic components detail is available with the supplementary material (doi:10.17632/499gx3pfxk.1).

## 5 Build instructions

This section describes the step-by-step instructions for the construction and assembly of the Flow-Batch controller (propulsion system and valve activator) in subSection 5.1, and the assembly of the Flow-Batch system in subSection 5.2.

The additional tools required for fabrication and assembly are:

•Soldering iron.
•Tin lead solder wire.
•Allen key set.
•Screwdrivers.
•Teflon Tape.
•Wires.

### 5.1 Hardware construction

#### 5.1.1 Electronic Part

**Fig. 7:**
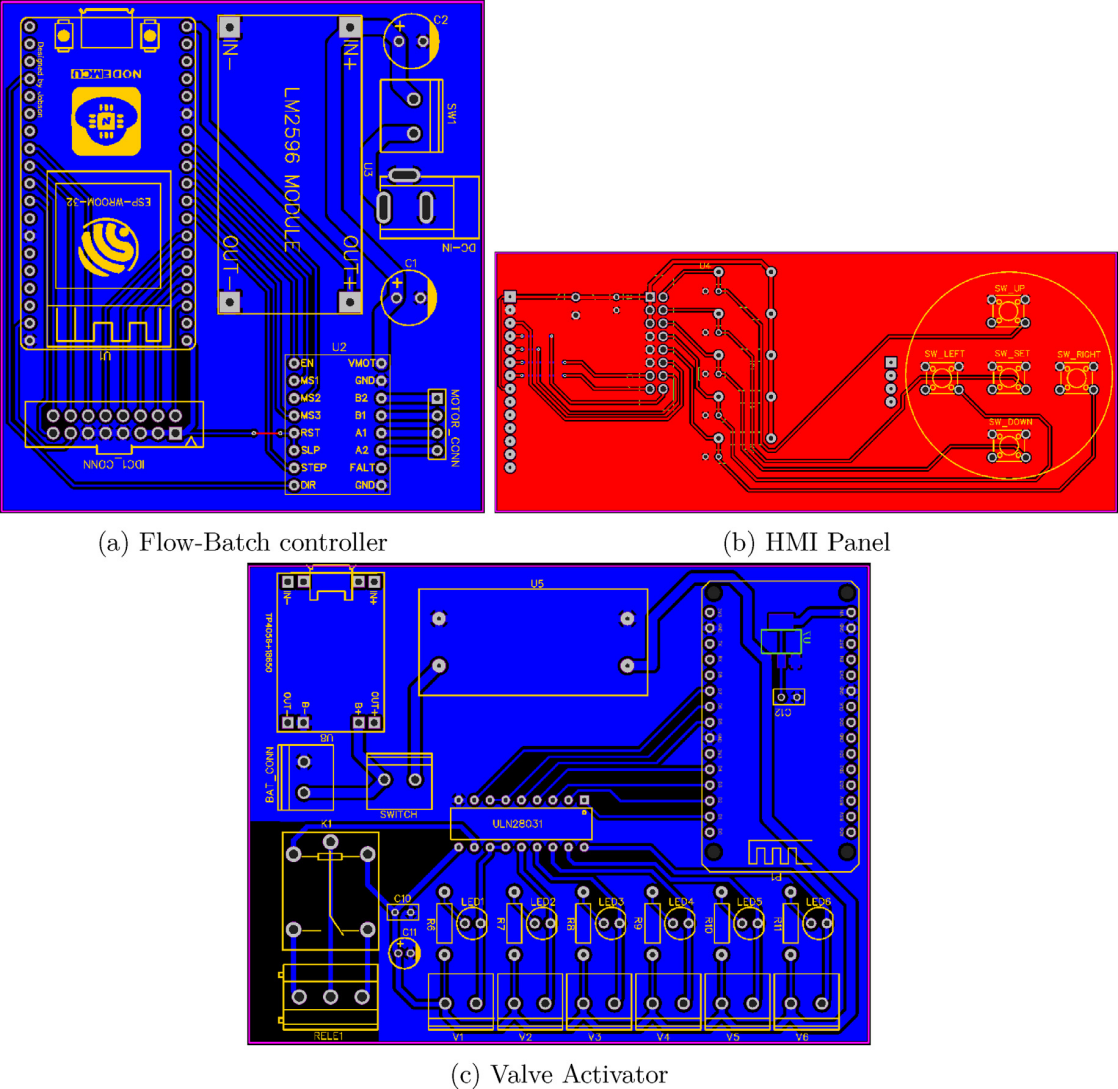
PCB designs from EasyEDA CAD software.
step 2: Solder the components on the PCB following the designator of each component in the bill of materials. In the case of microcontrollers (U1 and U5) and the DRV8825 driver (U2), it is recommended to solder female pin sockets instead of solder the components directly on the PCB. See Fig. 8

step 1: Manufacture the printed circuit boards (Part#1-Flow-Batch controller, Part#2-hmi panel and Part#3-valve actuator), see Fig. 7. This can be done on your own, or you can send the designs to a PCB manufacturing company.

**Fig. 8:**
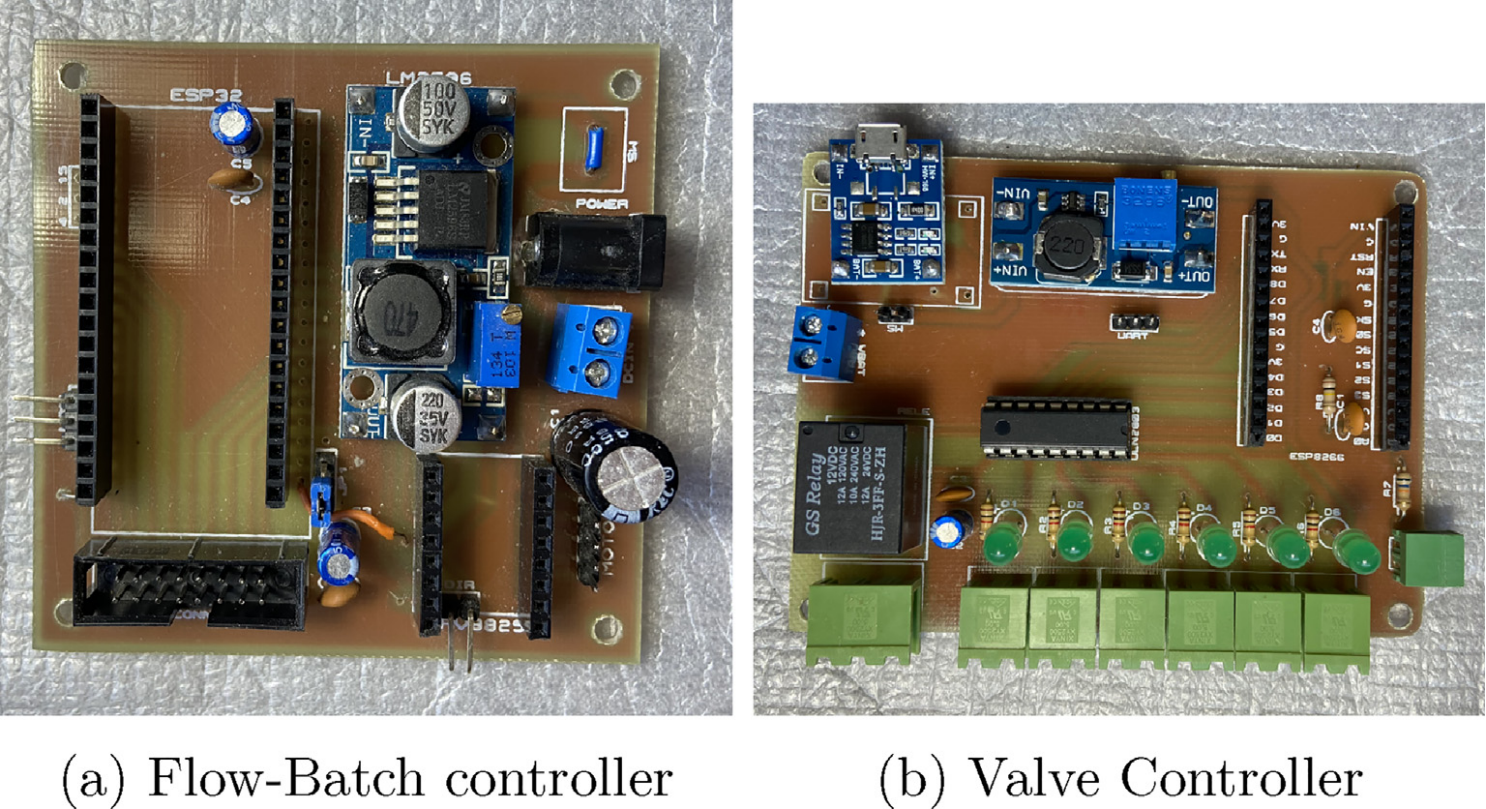
Finished PCBs.
step 3: Energize the board Part#1 with a 12 24VDC power adapter without connecting the microcontroller and driver to calibrate the output voltage of LM2596 module at 5V.
step 4: Energize the board Part#3 from microUSB cell charger in the TP4056 plug without connecting the microcontroller to calibrate the output voltage of MT3608 module at 12V.
step 5: Assemble the connector cable for the HMI panel using flat ribbon cable (Part#21) and IDC conectors (Part#20) as follow. See Fig. 9.

**Fig. 9:**
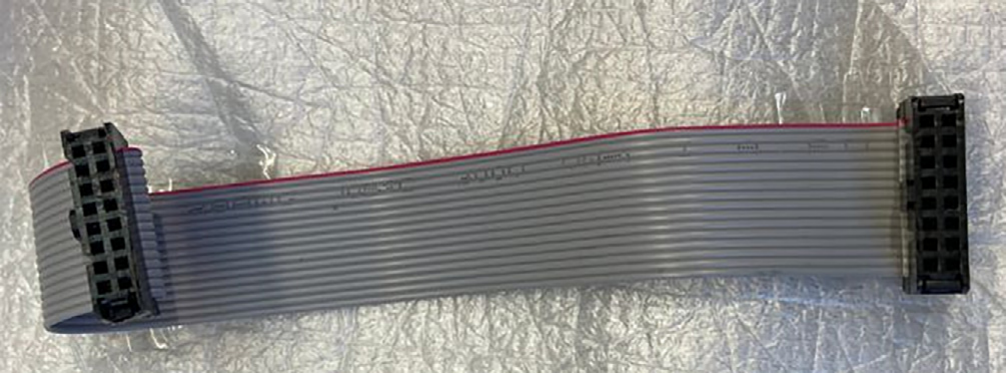
HMI Cable assembly.

#### 5.1.2 3D Printing and assembly

**Fig. 10:**
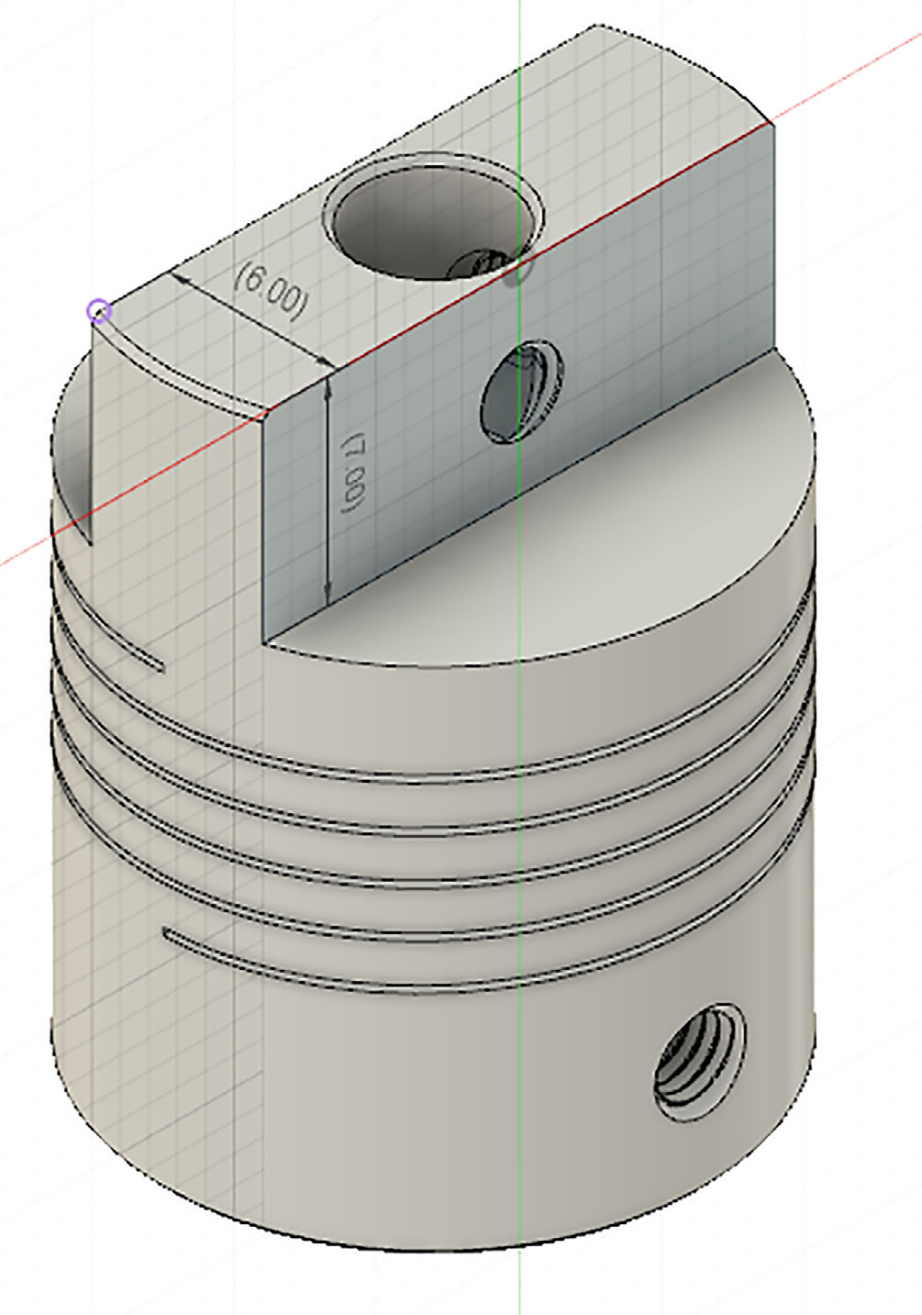
File the flexible shaft coupler.
step 3: Screw the flexible coupling (Part#26) to the shaft of stepper motor (Part#24) as can be seen in Fig. 11a, and then screw the motor to Part#4 using M2x8 screws, see Fig. 11b.

step 1: Print the pieces (Part#4, Part#5, Part#6, Part#7, Part#8 and Part#9) in the 3D printer.
step 2: File the flexible coupling (Part#26) according to the Fig. 10.

**Fig. 11:**
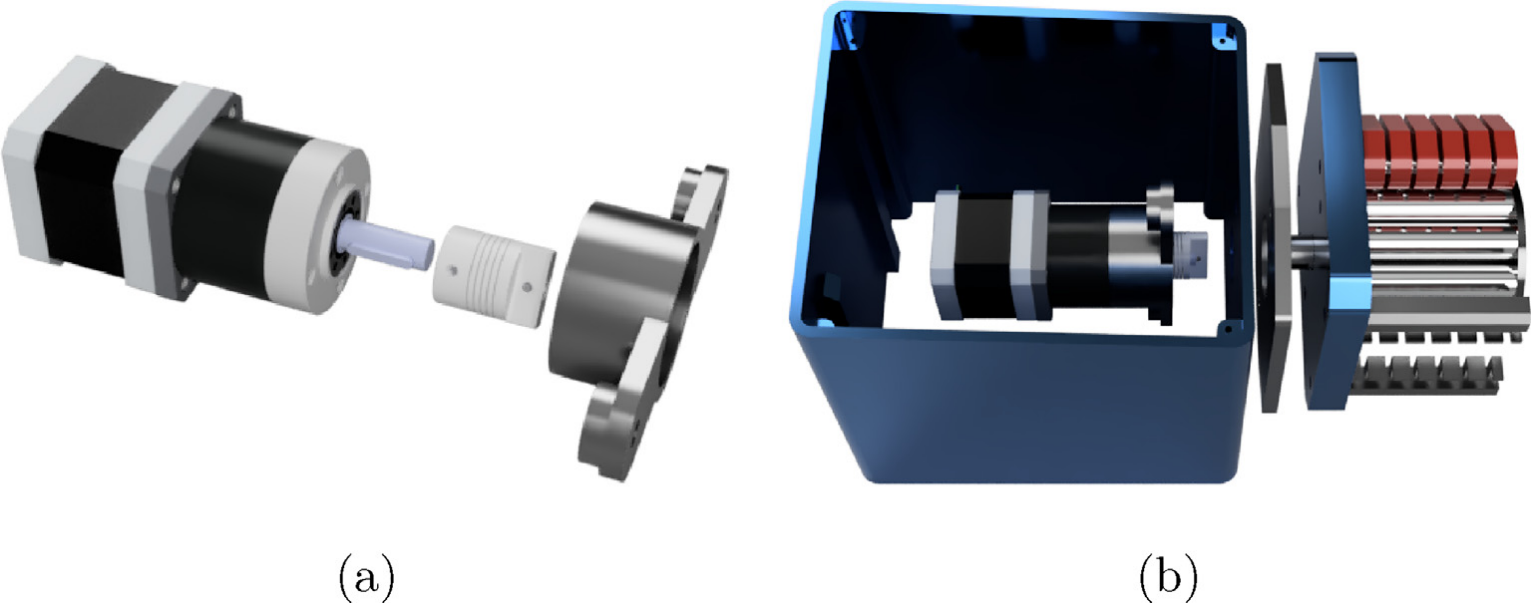
Stepper motor assembly.
step 4: Join the motor support (Part#4) to the cabinet (Part#6), using the supplement (Part#5) with M5x48 screws and M5 hexagonal nuts.
step 5: Screw the HMI panel PCB (previously placing the button printing Part#8) to Part#7 using M2x8 screws. See Fig. 12a

**Fig. 12:**
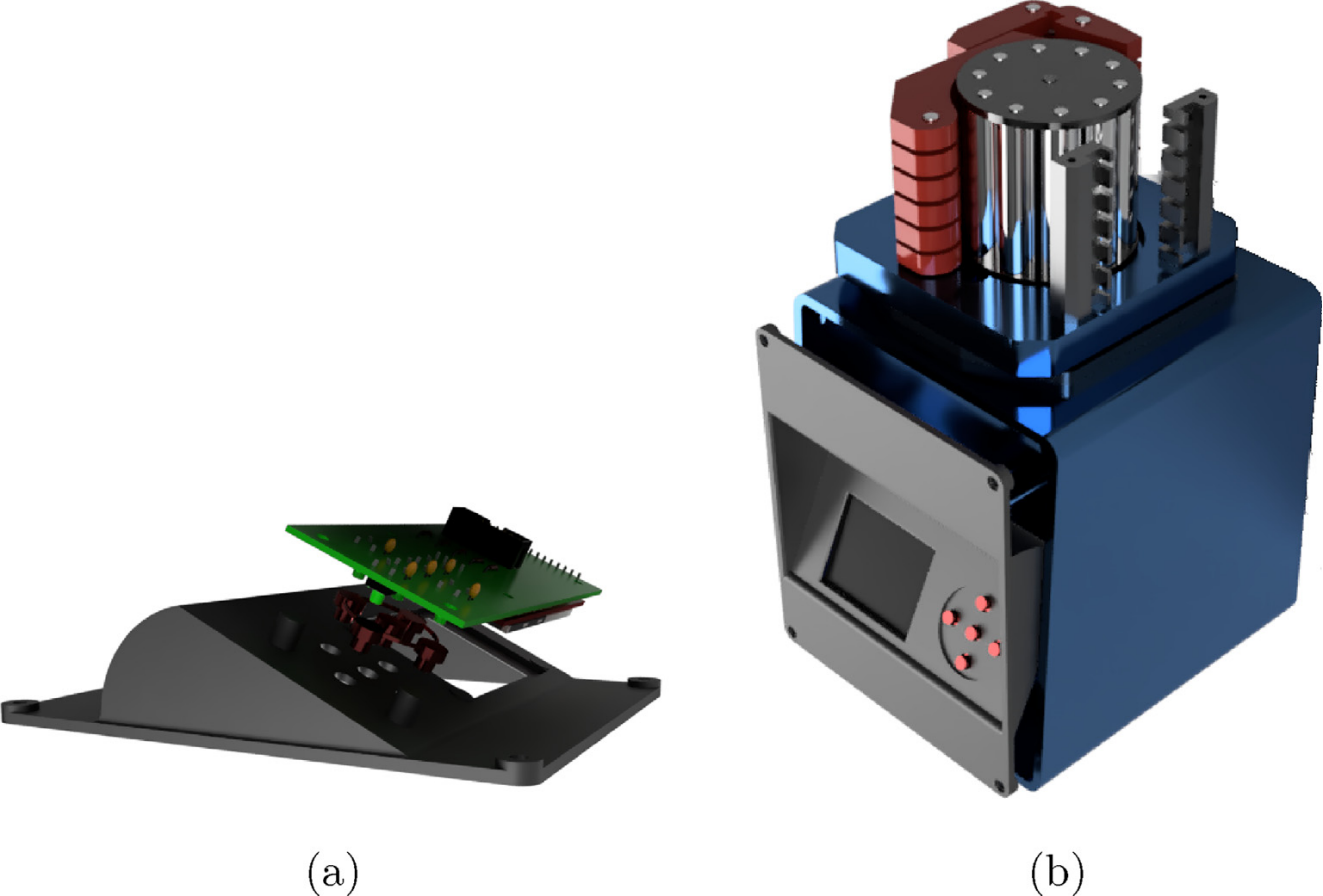
Front panel assembly.
step 6: Screw the HMI panel (Part#7) to the front of the cabinet (Part#6) M2x8 screws. See Fig. 12b
step 7: Screw the controller PCB (Part#1) to the cabinet (Part#6) and connect the cables of the HMI panel and the stepper motor.
step 8: Screw the rear panel (Part#9) using M2x8 screws and connect the ON/OFF key. See Fig. 13

**Fig. 13:**
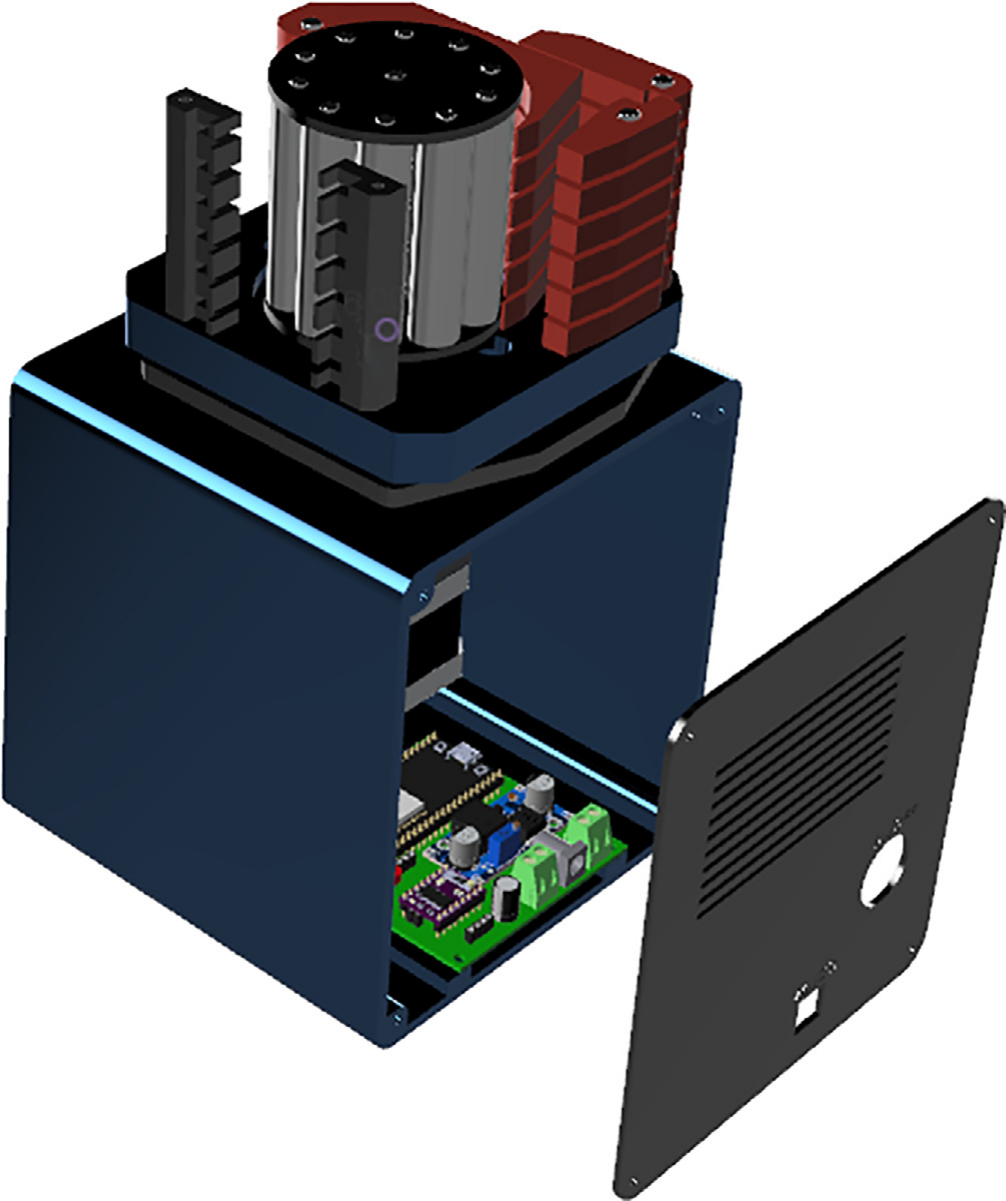
Rear panel assembly.
step 9: Print the pieces of the valve actuator case (Part#10 and Part#11) in 3D printer.
step 10: Screw the valve actuator PCB to the bottom case using M2x8 screws and connect the battery socket to terminal block P2.
step 11: Connect the ON/OFF key to terminal block P3 and screw the top case (Part#11). See Fig. 14

**Fig. 14:**
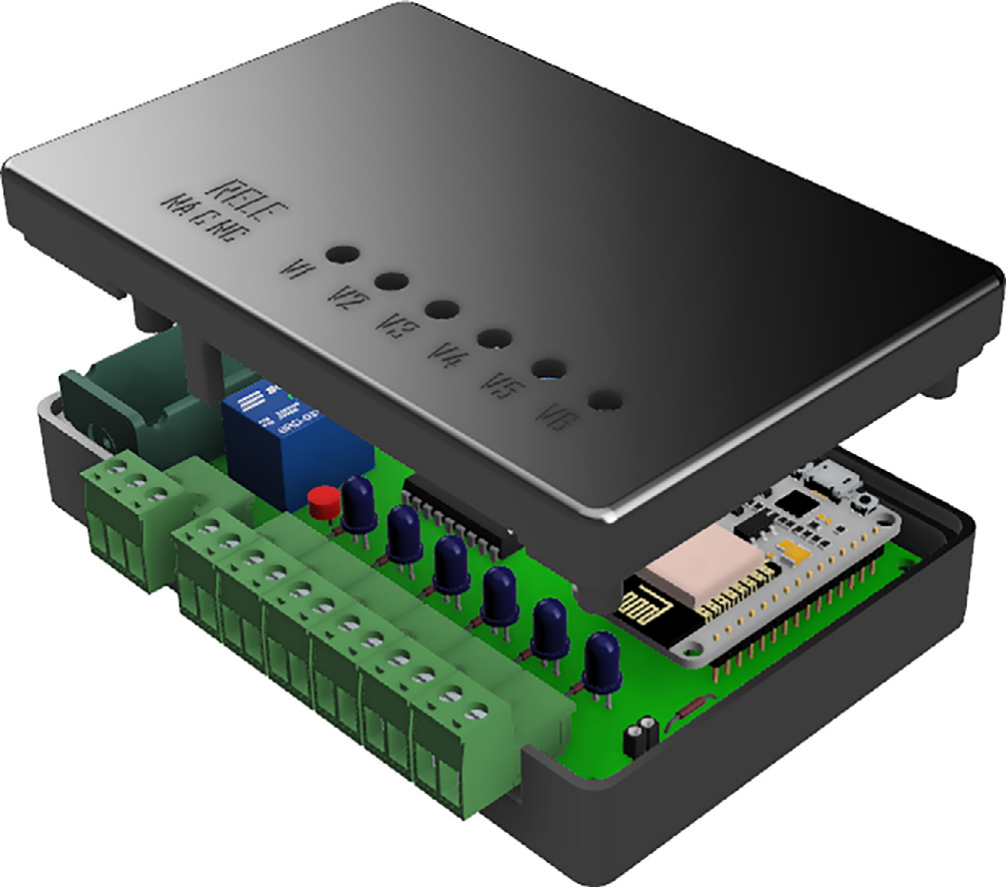
Valve activator assembly.

#### 5.1.3 Flashing the firmware of Flow-Batch main controller

step 1: Connect the ESP32 board to the PC through an USB port and open the Arduino IDE.
step 2: Load the Sketch “flowbatch_controller.ino” (Part#17).
step 3: On Tool Menu, select “ESP32 Dev kit V1.0” and then select the corresponding COM port.
step 4: Go to the Tool Menu and click on “ESP32 Sketch Data Upload”.
step 5: Compile and Upload the firmware to the microcontroller.

#### 5.1.4 Flashing the firmware of valve controller

step 1: Connect the ESP8266 board to the PC through an USB port and open the Arduino IDE.
step 2: Load the Sketch “valve_controller.ino” (Part#18).
step 3: On Tool Menu, select “NodeMCU V1.0 (ESP12-E module)” and then select the corresponding COM port.
step 4: Compile and Upload the firmware to the microcontroller.

#### 5.1.5 Printing the 3D parts of the chambers

**Fig. 15:**
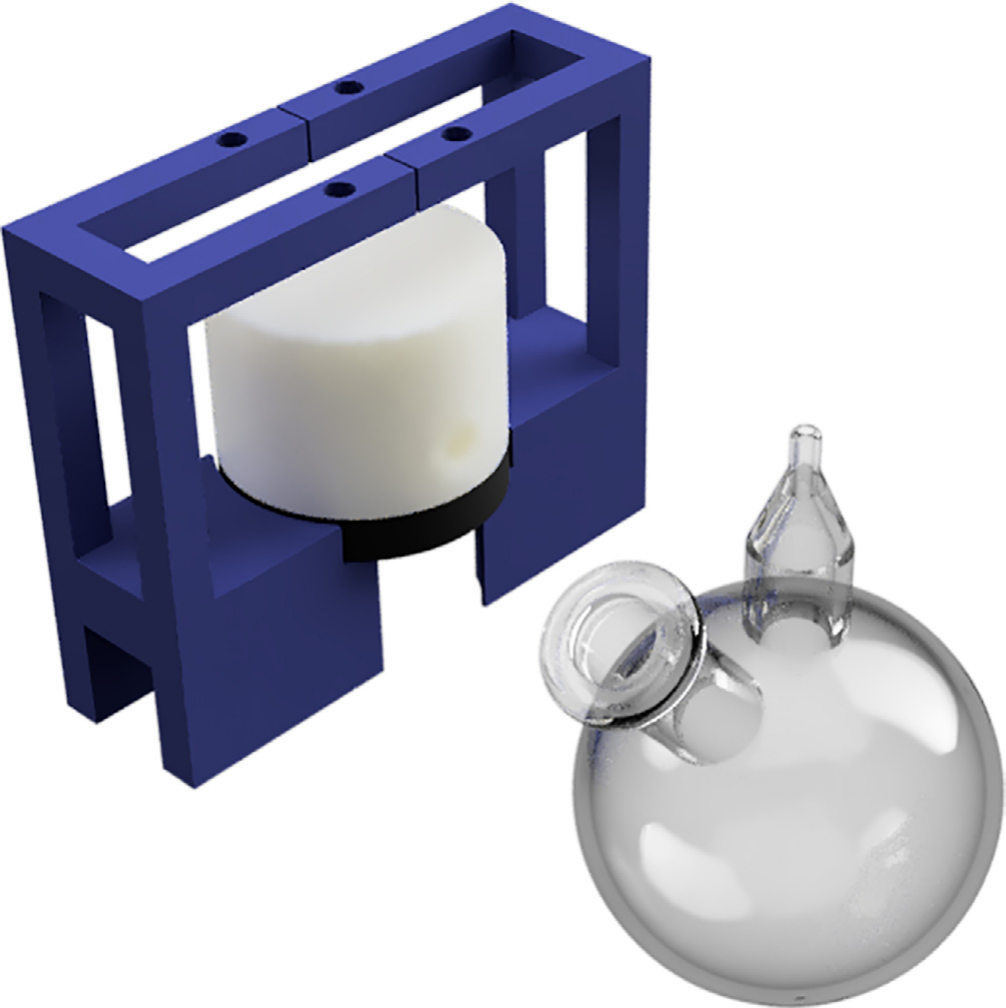
Fabrication and assembly of reaction and detection chambers.

step 1: Print the pieces needed for the assembly of the detection chamber (Part#14, Part#15 and Part#16) in 3D printer.
step 2: Manufacture the reaction chamber with glass, and the detection chamber from a cylinder of Teflon following the 3D model in “FlowBatch_Chambers.f3d” CAD file. See Fig. 15.

### 5.2 Assembly of the Flow-Batch system

**Fig. 16:**
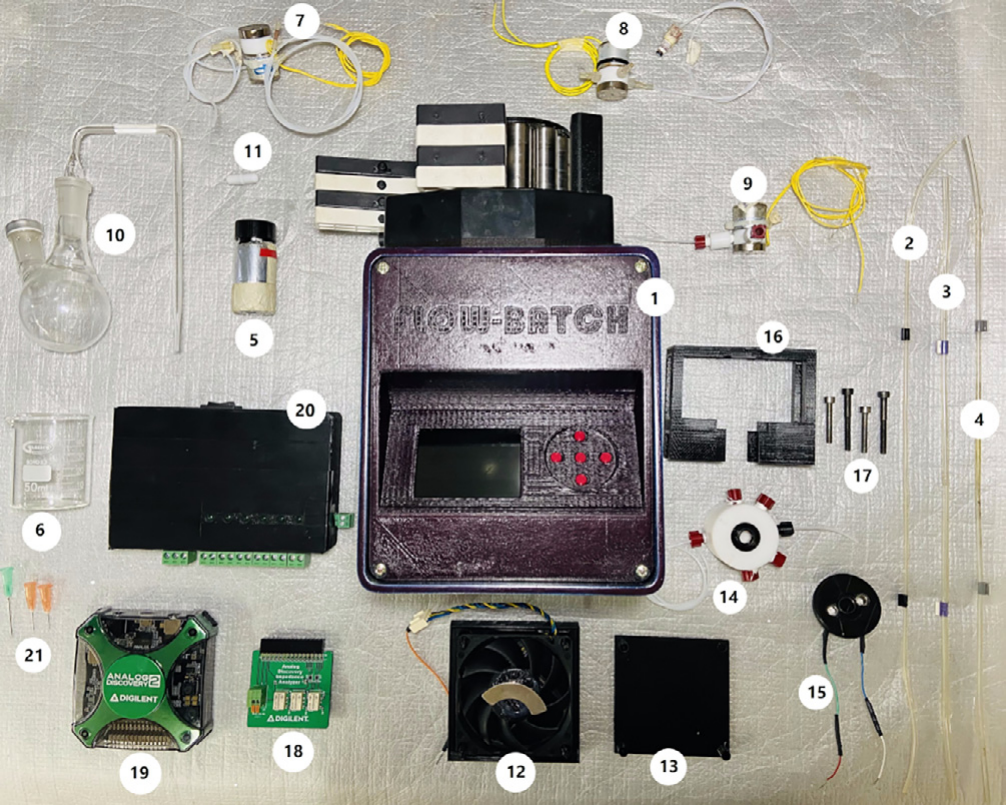
Photograph of the Flow-Batch System. **Piece #1)** Propulsion system. **Piece #2)** PVC tubing, ID: 0.76 mm. **Piece #3)** PVC tubing, ID: 2.06 mm. **Piece #4)** PVC tubing, ID: 1.30 mm. **Piece #5)** Glass tube holder coating with aluminium for NaBH_4_. **Piece #6)** Sample beaker. **Piece #7-9)** Solenoid valves. **Piece #10)** Glass flask. **Piece #11)** Magnetic stir bar. **Piece #12)** Cooler with magnet. **Piece #13)** 3D printed cooler top. **Piece #14)** Teflon chamber with o-ring. **Piece #15)** 3D printed chamber with leads for QCM contacts. **Piece #16)** 3D printed press of the detection chamber. **Piece #17)** Screws. **Piece #18)** Impedance Analyzer module. **Piece #19)** Analog Discovery 2. **Piece #20)** Valve actuator. **Piece #21)** Dispensing needles.

step 1: Place the tubings **Piece #2-4)** in the Flow-Batch **Piece #1)** header.
step 2: Place the **Piece #2)** input inside of **Piece #5)** and the **Piece #3)** input inside of **Piece #6)**. Place a dispensing needle **Piece #21)** at the end of each tubing that inputs **Piece #5)** and **Piece #6)**.
step 3: Leave the **Piece #4)** output to the atmosphere for the release of gases.
step 4: Place the **Piece #2)** output to the **Piece #7)** input.
step 5: Place the **Piece #3)** output to the input of the **Piece #8)**.
step 6: Assemble the reaction chamber (**Piece #10)**). Place the **Piece #11)** inside of **Piece #10)** and this last mount it on a flask support.
step 7: Assemble **Piece #12)** with **Piece #13)** and place this set under the reaction chamber (**Piece #10)**).
step 8: One of the outputs of the **Piece #7)** place inside of **Piece #5)** with the **Piece #2)**. The other output of the **Piece #7)** place in the input of the **Piece #10)**.
step 9: One of the outputs of the **Piece #8)** place inside of **Piece #6)** with the **Piece #3)**. The other output of the **Piece #8)** place in the input of the **Piece #10)**.
step 10: Assemble the detection chamber (**Pieces #14-17)**). Place the **Piece #15)** in the **Piece #16)** groove with the leads pointing down. Place the QCM over the **Piece #15)** o-ring. With extreme caution, place the **Piece #14)** over the **Piece #15)** (the QCM must be between the o-rings of the **Pieces #14-15)**). Insert the screws (**Piece #17)**) through the **Piece #16)** holes to adjust the **Piece #15)** against the **Piece #14)**.
step 11: The detection chamber input corresponds to the reaction chamber (**Piece #10)**) output, through a PVC tubing.
step 12: Connect the **Piece #14)** output to one of the **Piece #9)** inputs.
step 13: The second input of the **Piece #9)** corresponds to an air input.
step 14: The **Piece #9)** output corresponds to an input of **Piece #9)**.
step 15: Join the **Piece #18)** with the **Piece #19)** and connect the **Piece #19)** with a PC.
step 16: Connect the **Piece #15)** leads to the terminal block of **Piece #18)**.
step 17: Connections of **Piece #20)**. Through leads, connect each of the **Pieces #7-9)** to each of the terminal blocks of **Piece #20)**. Through WiFi, connect the **Piece #20)** to the **Piece #1)** see Fig. 16.

## 6 Operation instructions

### 6.1 Safety considerations

**Fig. 17:**
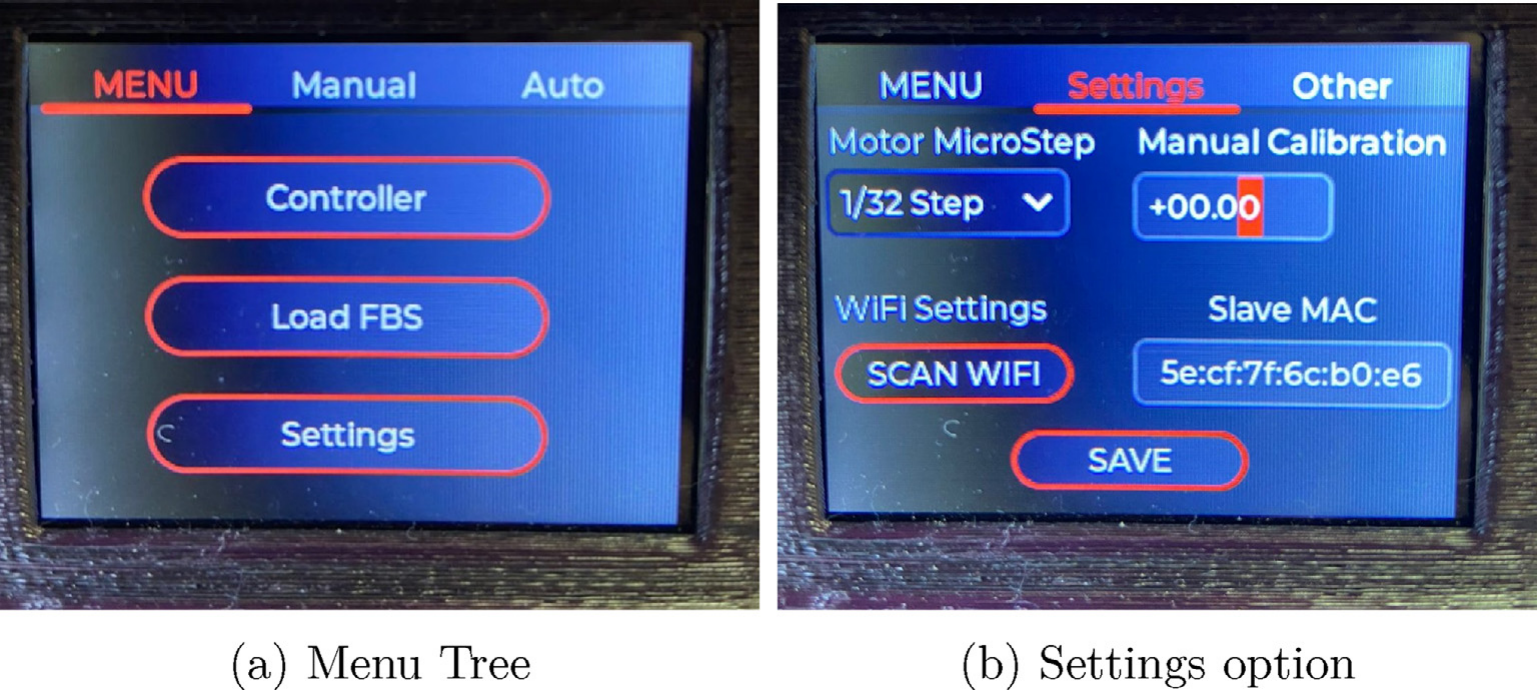
Menu.

The chemical reaction carried out in the glass chamber produces in the first place hydrogen (H_2_) and in case the sample contains arsenic, arsine gas is produced afterwards. As the hydrogen is highly explosive and the arsine gas is toxic, the system should be mounted under a laboratory fume hood to protect the person handling it. It must be ensured that there are no leaks in any connection between tubes, or in the reaction and detection chambers. In case of leaks it should be sealed using Teflon tape. see Fig. 17.

### 6.2 Flow-Batch controller operation

**Fig. 18:**
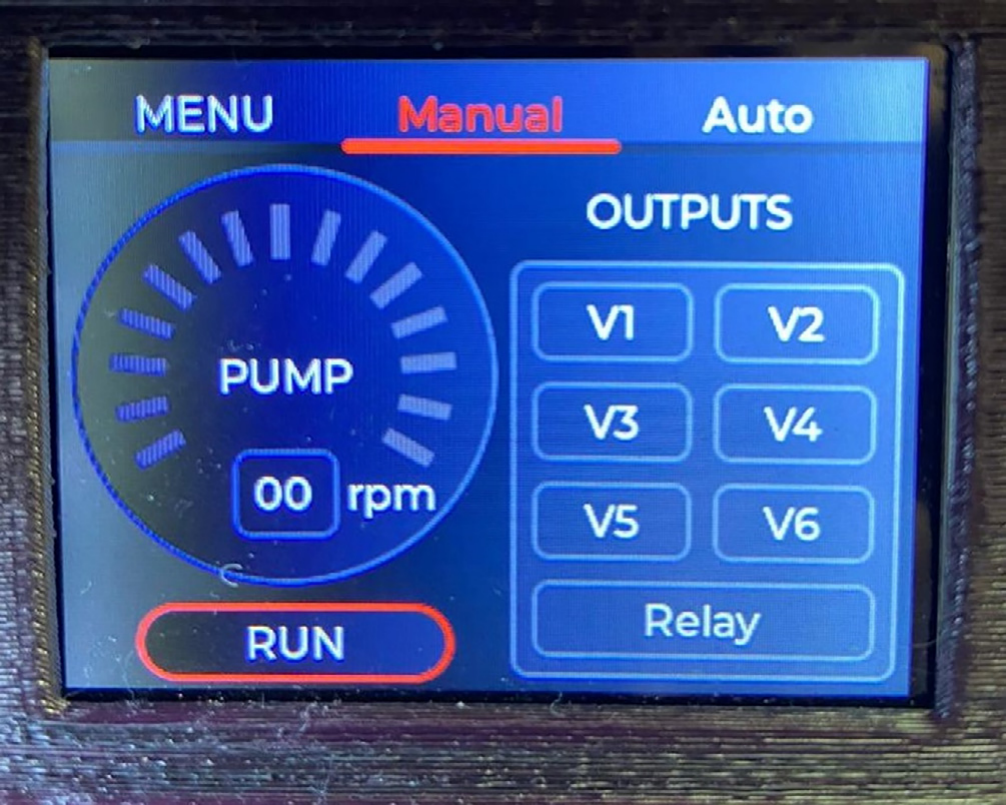
Manual mode.

The equipment has a simple and friendly user interface for easy handling by inexperienced users. Three options can be accessed from the menu: Controller, Load FBS and Settings. see Fig. 18.

The equipment has a manual operation mode where the speed of the pump and the activation of the outputs can be controlled manually through a screen that integrates all the commands.

**Fig. 19:**
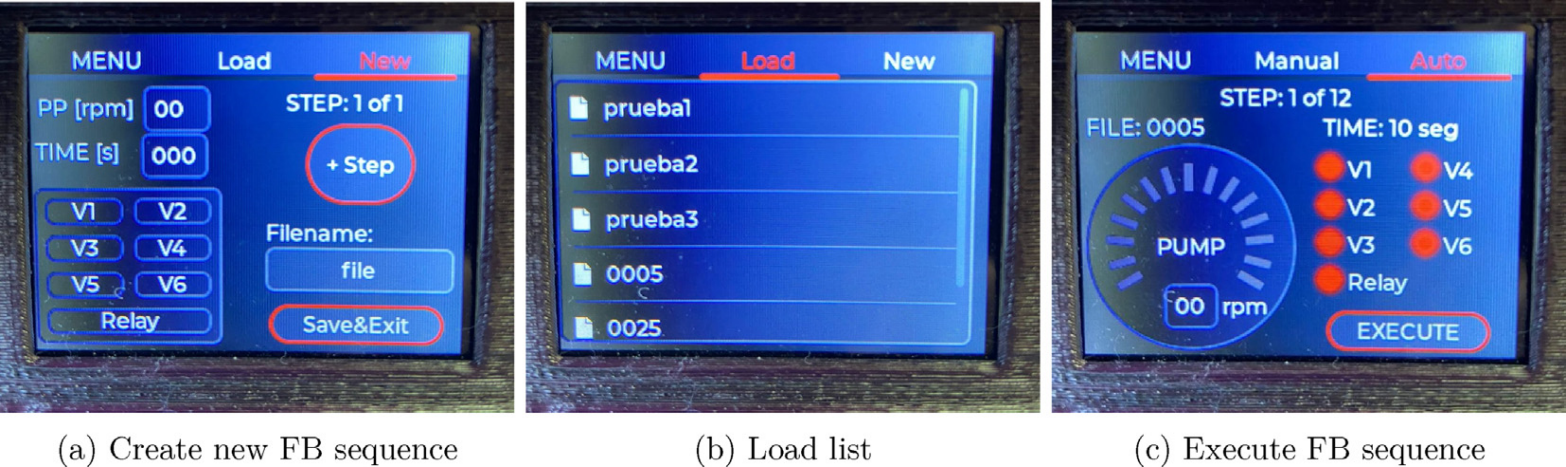
Steps for creating, loading and executing a FB sequence.

In automatic mode, the system executes a previously created sequence. Flow-Batch sequences are created and stored in the internal memory of the microcontroller in a json structure. For each step of a Flow-Batch sequence, it must be configured: the speed of the peristaltic pump (0 if it must be stopped), the step time in seconds, and the output that must be active. see Fig. 19.

## 7 Validation and characterization

In this section the characterization and validation of the system proposed is presented.

### 7.1 Characterization

**Fig. 20:**
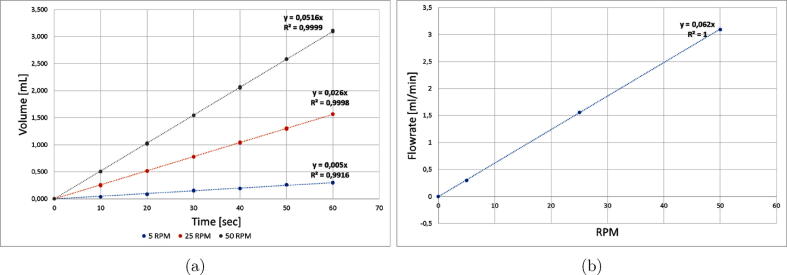
a) Flow-Batch displacement volume. b) Flowrate of Flow-Batch system.

The precision of fluid delivery is assessed by measuring the mass of water delivered as a function of time. A Shimadzu balance is used to measure with 3 decimals precision and a pumping tube (black-black) for the propulsion system. Fig. 20a displays the displacement volume for three different motor speed revolutions. Tests were conducted three times for each speed to check the repeatability of the experiments, especially in the flowrate. The pumps showed very good repeatability in all runs. The flowrate was 0.30 mL/min and 1.56 mL/min for 5 rpm and 25 rpm, respectively. The highest flowrate was 3.10 mL/min operated at 50 rpm of the motor speed (Fig. 20b). This experiment was carried out by toggling the output that controls a solenoid three-way valve.

The following equation express the volumetric flowrate (mL/min), *y*, as a proportional function of the angular velocity of the motor (rpm), *x*, where *m* is a constant:

y=mx

Fig. 20 show the calibration curves for the different Flow-Batch characterizations.

### 7.2 Validation of the system

The system was validated through a set of measures made with standard solution samples prepared in the lab. The experiment was set up under controlled conditions in a chemical laboratory. All reactions and measurements were made inside of a fume hood and using gloves and smocks for personal protection. An arsenic (As) stock solution (1000 μg/mL) was used and by dilution of this with distilled water, a set of standards solutions were prepared for testing, with dynamic range of arsenic concentrations 10 - 100 ppb. Before starting the analysis, the sample and ${\mathrm{NaBH}}_{4}$ solution were pumped and recirculated in the system. The solenoid valves V1 and V2 simultaneously switched on for 5 s to load the channels with the prepared solutions.

Afterwards, a series of steps were set in the Flow-Batch system to carry out the procedures automatically. V1 was switched on for 140 s and the sample was injected to the reaction chamber. Then, when V1 was switched off, S1 was switched on for magnetic stirring in the chamber. In the next step, the admittance analyzer was initialized and V2 and V3 were switched on for 810 s to inject the ${\mathrm{NaBH}}_{4}$ solution into the reaction chamber and for a continuous flow, respectively. Finally, data logging was stopped, S1 was switched off and then the wash cycle was done for the next samples. Table 1 shows the operation procedure that was used in the system for analyzing the three concentrations.

**Table 1:** Operation procedure.

Steps	Events	Time (s)	Pump setup (rpm)	Volume (mL)	Pump tube (mm id)	
1	Sample (V1)	140	30	10	2.06	
2	Magnetic Stirrer ON (S1)	-	-	-	-	
3	Initialize Admittance Analyzer	-	-	-	-	
	Sodium borohydride (V2)	810	3	2.5	0.76	
	Constant Flow (V3)	810	3	-	1.30	
4	Stop Admittance Analyzer	-	-	-	-	
5	Magnetic Stirrer OFF (S1)	-	-	-	-	
6	Wash cycle	-	-	-	-	

**Fig. 21:**
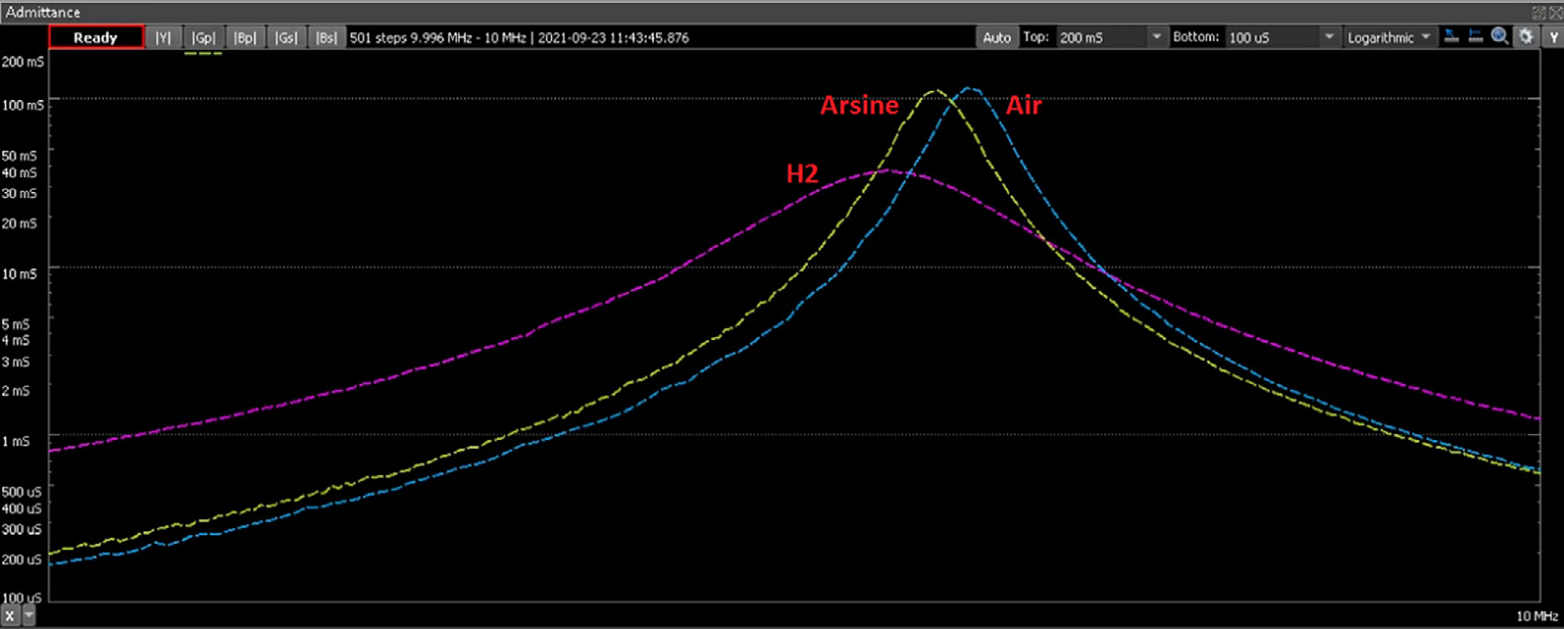
WaveForms plot of admittance analysis.

Fig. 21 shows the WaveForms 2D plot of admittance analysis of QCM where the difference between the density of air, hydrogen ($H_{2}$) and arsine can be perceived.

The process was followed for each solution prepared. They were placed in the sample beaker and the responses were registered and processed. Results are presented in Table 2 and the regression parameters R and the linear equation are computed.

(1) $$\begin{array}{cc} \Delta f & =0.8999x+189.59\\ R^{2} & =0.973 \end{array}$$

**Table 2:** Resonating frequency.

Concentration [ppb]	Δf [Hz]
10	191.9953
25	216.3655
50	240.0235
100	276.4456

**CRediT author statement**

**Julián Gutierrez**: Investigation, conceptualization, data curation, writing original draft preparation.

**Juan Pablo Mochen**: Investigation, conceptualization, data curation, writing original draft preparation.

**Gabriel Eggly**: Conceptualization, investigation, methodology, software, data curation, validation, writing original draft preparation.

**Marcelo Pistonesi**: Resources, supervision, project administration, funding acquisition, validation, writing review and editing.

**Rodrigo Santos**: funding acquisition, validation, visualization, writing original draft preparation, writing review and editing.

## Declaration of Competing Interest

The authors declare that they have no known competing financial interests or personal relationships that could have appeared to influence the work reported in this paper.
